# Treatment for thoracic ossification of posterior longitudinal ligament with posterior circumferential decompression: complications and managements

**DOI:** 10.1186/s13018-016-0489-4

**Published:** 2016-11-29

**Authors:** Baohui Yang, Yi Wang, Xijing He, Haopeng Li

**Affiliations:** 1 Department of Orthopaedics, The Second Affiliated Hospital of Xi’an Jiaotong University, Xi’an, Shaanxi 710004 People’s Republic of China; 2 Pediatric Intensive Care Unit, Xi’an Children’s Hospital, Xi’an, Shaanxi 710003 People’s Republic of China

**Keywords:** Thoracic vertebrae, Ossification of posterior longitudinal ligament, Posterior circular decompression, Pedicle-screw internal fixation, Complication

## Abstract

**Background:**

The complications and corresponding managements for patients with thoracic ossification of posterior longitudinal ligament (TOPLL) who were treated with posterior circumferential decompression have not been systematically summarized yet.

**Methods:**

Twenty-one patients with TOPLL who received posterior circumferential decompression between February 2010 and December 2014 were retrospectively reviewed. The patients’ basic characteristics, surgical duration, intraoperative blood loss, Japanese Orthopaedic Association (JOA) scores, and intraoperative and postoperative complications, and the adopted managements were summarized.

**Results:**

The patients were averagely aged 52.1 ± 8.3 (range 32–67) years and included 10 males and 11 females. The mean operation time was 4.0 ± 0.9 (range 2.5–6) h and blood loss was 1619 ± 704 (range 800–4000) ml. Patients were followed up for 24.5 ± 1.2 (range 12–36) months. The average JOA score of patients was significantly elevated from 4.5 ± 1.4 (preoperative) to 7.4 ± 2.4 (*P* < 0.001, mean recovery rate 57.73%) on the second postoperative day and 7.8 ± 2.2 (*P* < 0.001, mean recovery rate 60.36%) at the final follow-up visit, respectively. There were 23 cases of complications that occurred in 12 patients, including 10 cases of intraoperative hemorrhage, 5 of cerebrospinal fluid leakage, 4 of intercostal nerve palsy, 3 of neurological deterioration, and 1 of superficial infection. After the corresponding treatment, these complications were recovered during the follow-up except 1 case of postoperative neurological deterioration did not exhibit improvement.

**Conclusions:**

Posterior circumferential decompression is effective for TOPLL but causes complications which need to be proactively prevented and treated. If treated properly, most complications can be recovered with satisfactory outcomes.

## Background

Thoracic ossification of posterior longitudinal ligament (TOPLL) is not common in clinical practice, with an incidence of approximate 0.8% [1]. TOPLL is often progressively aggravated once discovered, which can result in spinal cord injury and even paralysis [2]. Surgical decompression is the only effective option for the treatment. Due to the anatomy of the blood supply, the thoracic spinal cord cannot be reached by the great radiculomedullary artery and thus belongs to an ischemic area. Correspondingly, spinal cord ischemia and ischemia-reperfusion injury during operations aggravate the symptoms. It was reported that neurologic deficit and cerebrospinal fluid leakage were the most common complications post the surgical decompression [3–5]. Several surgical procedures were employed in the treatment of TOPLL, but how to choose the optimal surgical plan still remains controversial [6]. Anterior decompression is an ideal treatment approach for TOPLL, while it is technically demanding with a high rate of complications [7, 8]. Posterior decompression for TOPLL is less technically demanding and also effective [9, 10], while it provides indirect decompression and thus leads to relatively lower clinical recovery rate compared with other surgical approaches (e.g., anterior or circumferential decompression) [9]. Circumferential decompression through posterior approach (i.e., one-stage posterior circumferential decompression) becomes a more effective treatment option [3, 4, 11, 12]. It was shown that circumferential decompression achieved more recovery rate and fewer postoperative complications (e.g., paralysis) than posterior decompression [13]; however, this procedure also accompanies with a relatively high rate of complications. For example, Takahata et al. reported posterior circumferential decompression caused dural tear (in 40% cases), deep infection (in 10% cases), and postoperative neurologic deterioration (in 33% cases) in patients with TOPLL [6]. Li et al. demonstrated paralysis, leakage of cerebrospinal fluid, and urinary tract infection occurred after the posterior circumferential decompression for TOPLL [13]. In addition to neurologic deficit and cerebrospinal fluid leakage, other complications for posterior circumferential decompression in the treatment of TOPLL included infection and subcutaneous fluid collection [4]. Thus far, however, the complications from posterior circumferential decompression for TOPLL and corresponding management measures have not been systematically summarized yet.

Here, we retrospectively reported the intraoperative and postoperative complications as well as the causes and corresponding management measures in 21 patients with TOPLL who received posterior circumferential decompression and pedicle-screw internal fixation between February 2010 and December 2014 at The Second Affiliated Hospital of Xi’an Jiaotong University (Xi’an, China).

## Methods

### Patients

Twenty-one patients with TOPLL who received posterior circumferential (360°) decompression and pedicle-screw internal fixation between February 2010 and December 2014 at The Second Affiliated Hospital of Xi’an Jiaotong University (Xi’an, China) were retrospectively reviewed. Patients with obvious TOPLL and with available follow-up data were included. Patients combined with spinal tumors or tuberculosis and those intolerant to surgery were excluded. Patients were diagnosed by the preoperative routine X-ray, computed tomography (CT), and magnetic resonance imaging (MRI) examinations. The study was in accordance with the Declaration of Helsinki and was approved by Ethics Committee of The Second Affiliated Hospital of Xi’an Jiaotong University (Xi’an, China). Written informed consent was obtained from all patients.

### Surgical procedure

**Fig. 1:**
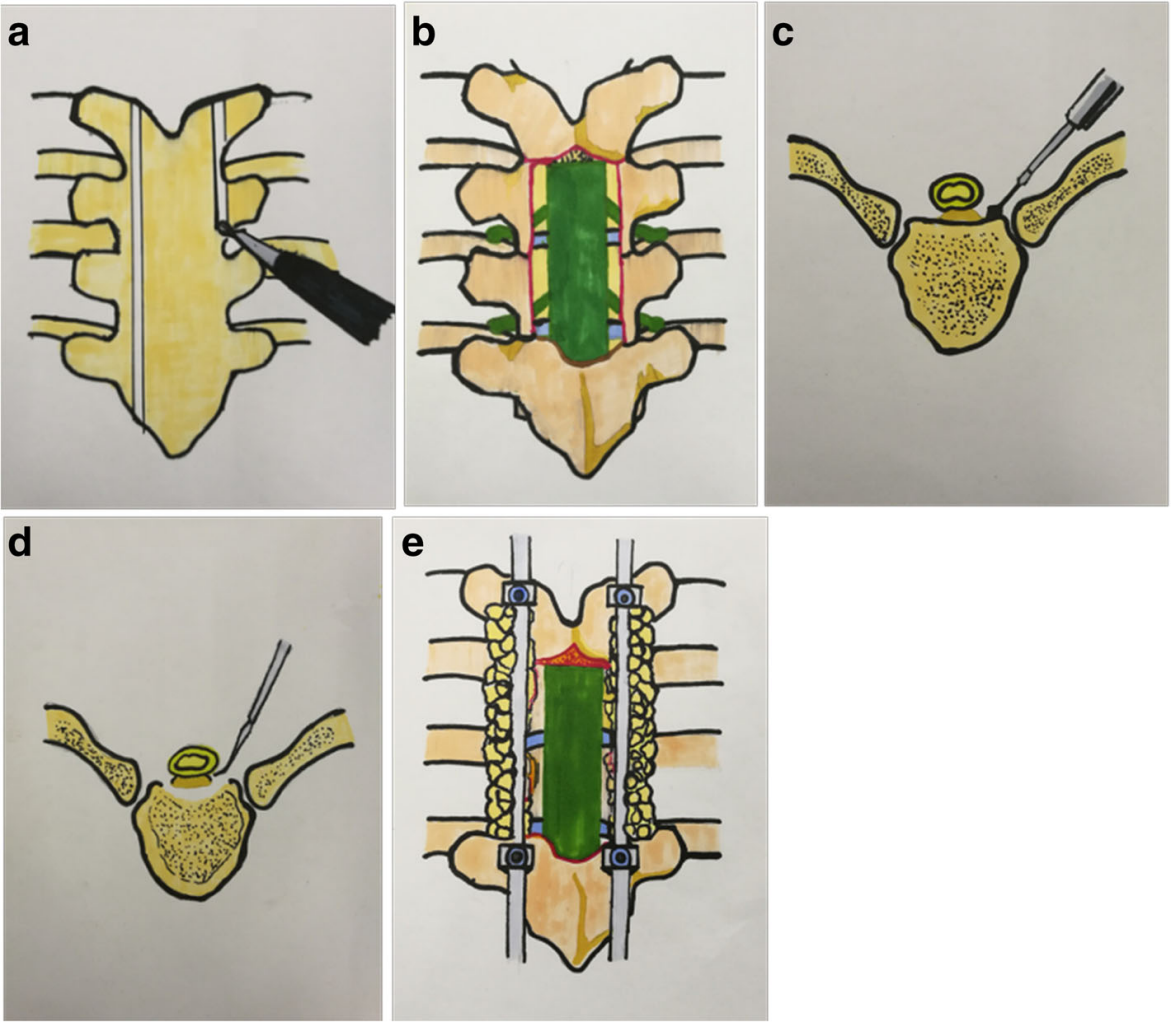
The illustration of the surgical procedure. **a** The lamina was cut along the axis of the both sides of the small joints using a high-speed burr, like “lifting the lid”. **b** The dura mater and PLL at the front and tissues within the vertebral column was exposed. **c** A burr was used to remove 1/3 of the back of the injured vertebral body. **d** A nerve dissector was used to separate the ossified PLL or make it suspending. **e** The resected posterior accessory bone was used as a graft to implant on the outer posterior side for fusion and internal fixation

**Fig. 2:**
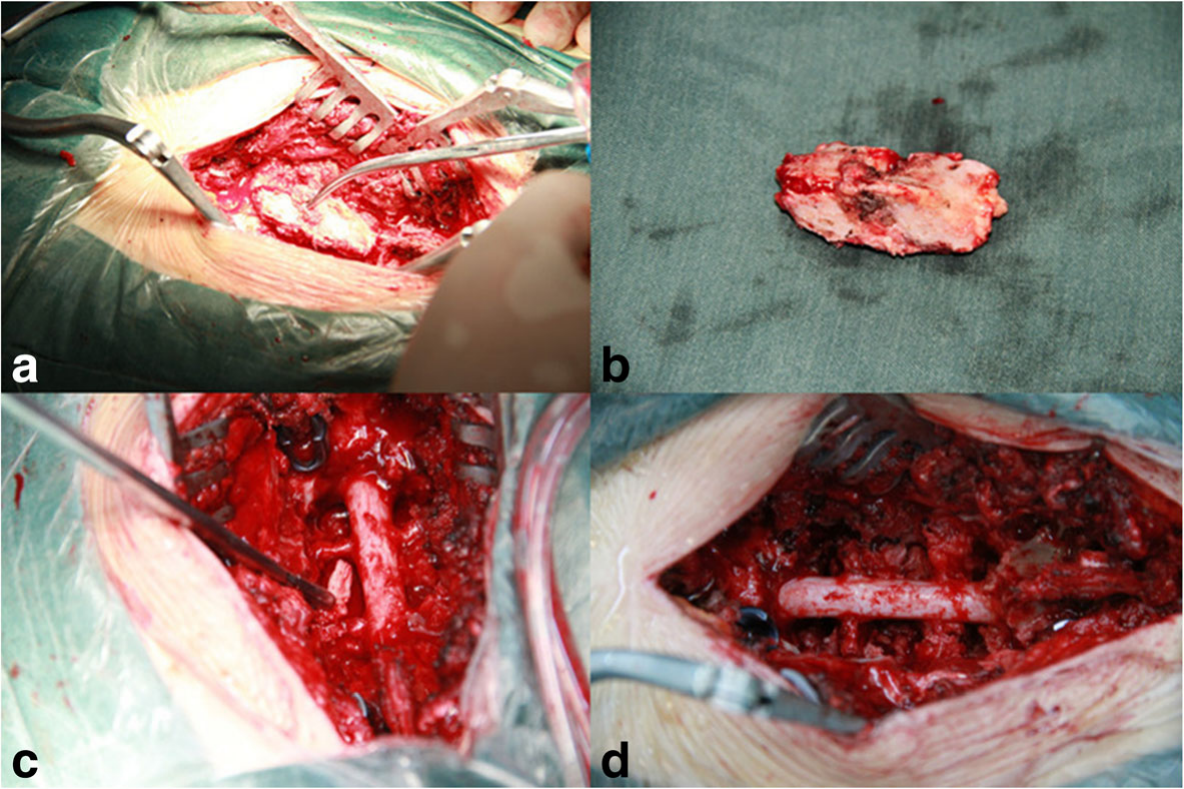
Surgical pictures of intraoperative resection of the PLL. **a** Complete removal of the lamina for decompression from the posterior like “lifting the lid” method. **b** Completely removed rear lamina. **c** Resection and removal of the posterior ligament ossified at the front. **d** Complete resection of the PLL

**Fig. 3:**
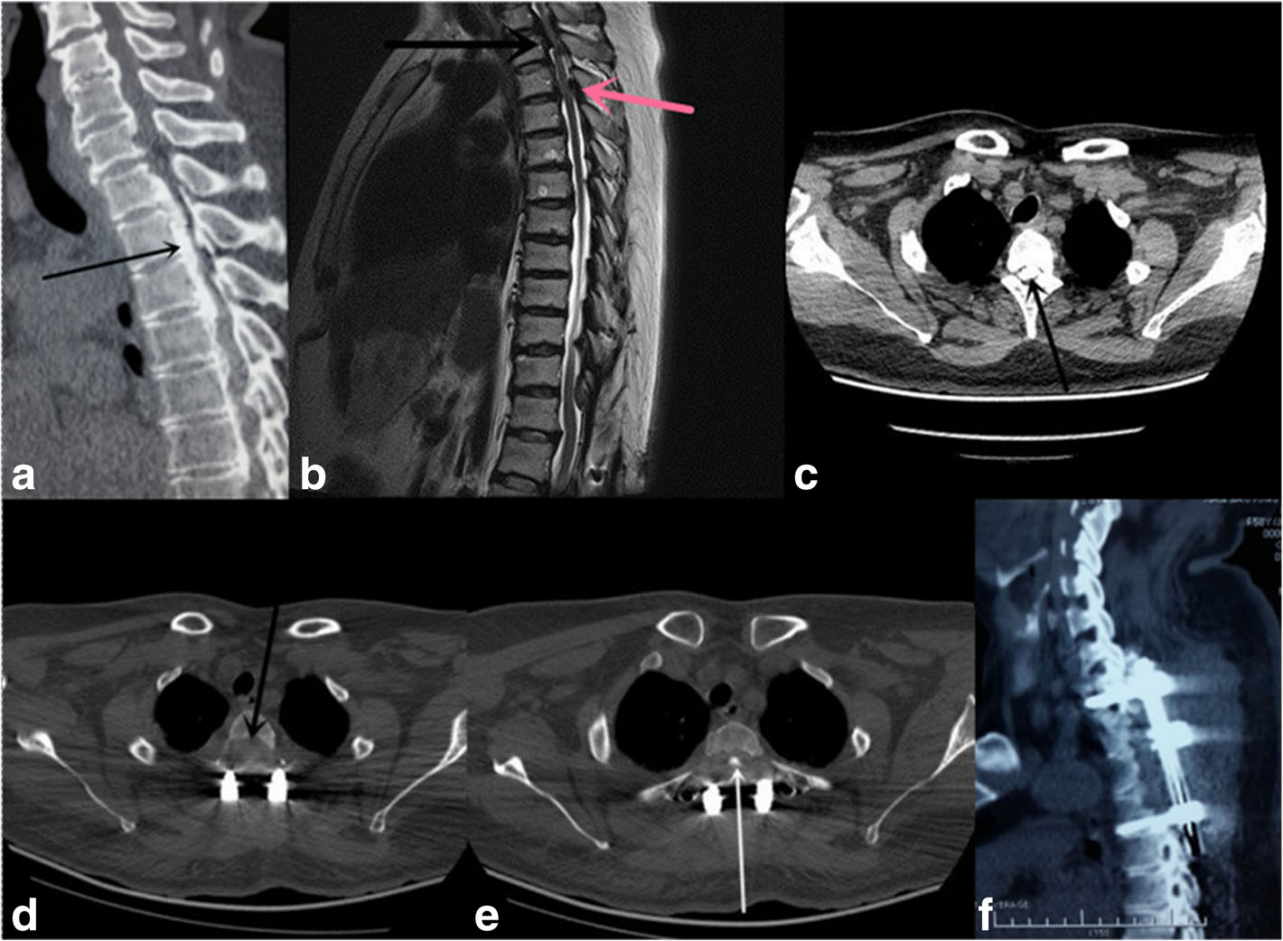
Pre- and post-operative imaging of thoracic OPLL with stenosis in a 65-year-old female patient (patient #18). **a** Preoperative CT showed OPLL at T1-4 (*arrow, sagittal view*). **b** Preoperative MRI showed spinal cord compression at segments T1-4 and yellow ligament hypertrophy at T3 (*arrow, sagittal view*). **c** Preoperative CT image showed ossification of PLL at the T2 level (*arrow, cross-sectional view*). **d** Postoperative CT image showed complete removal of the PLL at the T2 level (*arrow, cross-sectional view*). **e** Postoperative cross-sectional CT image showed a suspending PLL at the T3 level (*arrow, cross-sectional view*). **f** Postoperative CT image showed removal of 1/3 of the T1-4 vertebral bone and PLL (*sagittal view*)

Under general anesthesia, patients were placed in a prone position, with the abdomen exposed. Lesions were located by X-ray examination with a C-arm, and the region to receive decompression was determined. Incision was started at the lesion segment and reached 1 to 2 spinal segments above and below the lesion segment, respectively. The skin subcutaneous layer and fascia were then incised sequentially, and the paraspinal muscles were stripped to expose the spinous process, lamina, and facet. In the upper and lower segments for decompression, pedicle screws were implanted for vertebral fixation. Using a high-speed burr, the lamina was cut along the axis of the both sides of the small joints, like “lifting the lid” (Figs. 1a and 2a, b). The dura mater and posterior longitudinal ligament (PLL) at the front and tissues within the vertebral column were thus exposed (Fig. 1b). One side was prefixed with a connecting rod, and the contralateral side was thus removed the remaining lamina and vertebral pedicle spanned by the PLL of the facet joint with a burr or a rongeur. The nerve root and segmental vessels should be carefully separated and protected. The rib vertebral and costotransverse joints were retained, and the rib did not need to be resected. The other side was then operated in the same way. After the exposure of the dura mater and PLL at the front, a burr was used to remove 1/3 of the back of the injured vertebral body from the both sides of the posterior wall of the vertebral column (Fig. 1c), which was then passed through the two sides. A thin layer of the cortical bone in front of the dura mater was retained. The PLL and dura mater were carefully explored and separated, and the ossified PLL was resected and removed from one side by a nerve dissector (Figs. 1d and 2c, d). If the ossified PLL was relatively large and adhered to the dural adhesions, it was not required to be completely resected and was left suspending instead (Figs. 1d and 3e). The rod linking the pedicle screws was then reinstalled and connected, and the resected posterior accessory bone was used as a graft to implant on the outer posterior side for fusion and internal fixation (Fig. 1e). Fibrin glue or a gelatin sponge was used to cover the dura, and a drainage tube was placed in the wound. Lastly, the wound was closed by layer-by-layer suturing.

### Intraoperative and postoperative management

Patients were intravenously administered 40 mg methylprednisolone during the operation. After surgery, patients received routine hormone treatment (10 mg/day dexamethasone for the first 3 days and 5 mg/day for the 4th to 6th days) to decrease the spinal cord edema and inflammation. Simultaneously, neurotrophic drugs (0.5 mg/day mecobalamin for some days depending on the disease condition) were used to promote the nerve recovery. In general, 2 days later after surgery, the drainage tube was removed. Two weeks later, patients were encouraged to make physical movements while wearing an orthosis for ambulation.

Neurologic function was monitored by routine examination of intraoperative somatosensory evoked potential. Hemorrhage was defined as blood loss >1500 ml. Intercostal nerve palsy, neurologic deterioration, and infection were also recorded. Complication in this study was defined as intraoperative hemorrhage, cerebrospinal fluid leak, intercostal nerve paralysis, neurologic deterioration, and infection. Patients with hemorrhage were treated with autologous or allogeneic transfusion of red blood cells, or with supplementation of plasma and colloidal components. For patients with cerebrospinal fluid leakage, the drainage tube was extubated 1 to 2 weeks later after the surgery instead of 2 days later as usual. Patients with intercostal nerve palsy were administered with dehydrating agents (mannitol, 125 ml/time, twice daily, intravenous drip), hormones (dexamethasone), or neurotrophic drugs (mecobalamin). Patients with postoperative neurological deterioration were provided with postoperative treatments of neurotrophic drugs (mecobalamin), hormone therapy (dexamethasone), and rehabilitation-oriented exercise (including muscle training, balance training, and body coordination training). Patient with incision-caused superficial infection underwent dressing change and debridement.

### Clinical evaluation

Modified Japanese Orthopaedic Association (JOA) scores (0–11 points) [14] were adopted to evaluate the improvement of neurologic function and recovery rate. Recovery rate = [(postoperative JOA score−preoperative JOA score)/(11−preoperative JOA score)] × 10%.

### Statistical analysis

Quantitative data were presented as mean ± standard derivative and were analyzed using SPSS 11.0 statistical software (SPSS Inc., Chicago, IL, USA). A pair *t* test was used for the statistical comparison between the postoperative and preoperative JOA scores. *P* < 0.05 was considered as statistically significant.

## Results

There were 21 patients, including 10 males and 11 females, with a mean age of 52.1 ± 8.3 (range 32–67) years. There were 6 cases of local ossification, 3 of mixed ossification, 7 of continuous ossification, and 5 of segmental ossification (Table 1). Six cases were combined with ossification of the yellow ligament, and 6 with ossification of the ligamenta flava. Regarding the lesion area, 13 cases were upper thoracic vertebra-involved and 8 were mid-lower thoracic vertebra-involved (Table 1).

**Table 1:** Characteristics and treatment outcomes of patients with TOPLL

Patient number	Age (years)	Gender	Type of TOPLL	Involved level	Operation time (h)	Blood loss (ml)	Complications	JOA before surgery	JOA^1^/RR	JOA^2^/RR
1	61	M	Local	T4	4	1600	Hemorrhage	4	7/42.86%	8/57.14%
							Cerebrospinal fluid leakage			
2	49	M	Local	T8	5	2100	Hemorrhage	5	9/66.67%	10/83.33%
3	52	F	Continuous	T1-3	6	4000	Hemorrhage	7	10/75.0%	10/75.0%
							Superficial infection			
4	55	F	Mixed	T5, T7-8	3.5	1700	Hemorrhage	4	2	5
							Intercostal nerve palsy			
							Neurological deterioration			
5	44	M	Mixed	T1-2, T3	4.5	1400		3	7/50.0%	8/62.5%
6	67	M	Continuous	T2-5	4	1300		3	9/75.0%	9/75.0%
7	48	F	Segmental	T3, T4	3	1200		6	9/60.0%	10/80.0%
8	53	F	Continuous	T1-4	4.5	2500	Hemorrhage	5	4	6
							Cerebrospinal fluid leakage			
							Intercostal nerve palsy			
							Neurological deterioration			
9	61	M	Local	T11	3	1200		4	9/71.43%	9/71.43%
10	49	F	Segmental	T5, T7	3.5	1400	Intercostal nerve palsy	4	8/57.14%	8/57.14%
11	56	M	Segmental	T2, T3	4.5	1600	Hemorrhage	3	2	2
							Neurological deterioration			
12	45	F	Continuous	T6-8	5	2300	Hemorrhage	3	7/50.0%	8/62.5%
							Cerebrospinal fluid leakage			
13	44	M	Local	T2	3	800		5	7/33.33%	6/16.67%
14	47	F	Continuous	T8-11	3.5	1300		4	8/57.14%	6/28.57%
15	32	M	Mixed	T1, T2-4	3	1000	Cerebrospinal fluid leakage	3	7/50.0%	7/50.0%
							Intercostal nerve palsy			
16	57	F	Local	T4	3.5	1000		6	9/60.0%	9/60.0%
17	47	M	Segmental	T4-6	4.5	1800	Hemorrhage	5	9/66.67%	10/83.33%
							Cerebrospinal fluid leakage			
18	65	F	Continuous	T1-4	4	1400		2	4/22.22%	4/22.22%
19	56	F	Continuous	T2-4	4.5	1900	Hemorrhage	5	9/66.67%	9/66.67%
20	49	M	Segmental	T5-7	4	1600	Hemorrhage	6	9/60.0%	9/60.0%
21	56	F	Local	T3	2.5	900		7	10/75.0%	10/75.0%
Mean ± SD	52.1 ± 8.3				4.0 ± 0.9	1619 ± 704		4.5 ± 1.4	7.4 ± 2.4*/57.73%	7.8 ± 2.2**/60.36%

*M* male, *F* female, *T* thoracic, *JOA* ^1^ JOA score at 2 days after the surgery, *JOA* ^2^ JOA score at the last follow-up visit, *RR* recovery rate

*P < 0.001 compared with JOA before surgery

All patients underwent a one-stage posterior circumferential decompression with a mean operation time of 4.0 ± 0.9 (range 2.5–6) h, a mean blood loss of 1619 ± 704 (range 800–4000) ml, and a mean follow-up duration of 24.5 ± 1.2 (range 12–36) months. The average JOA score was significantly elevated from preoperative 4.5 ± 1.4 points to 7.4 ± 2.4 points (*P* < 0.001) on the second postoperative day and to 7.8 ± 2.2 points (*P* < 0.001) at the final follow-up visit, respectively. The latter two postoperative JOA scores were not significantly different (*P* = 0.59) (Table 1). The patients showed a neurologic recovery rate of 57.73% on the second day after the surgery and 60.36% at the final follow-up visit. All patients except patient 11 were improved the neurologic function at the final follow-up visit. Preoperative CT and MRI images of a typical case of a 65-year-old female patient with TOPLL at T1-4 levels were shown in Fig. 3.

Totally, there were 23 complications developed in 12 patients. Averagely, the intraoperative blood loss was 1619 ± 704 (range 800–4000) ml, and particularly, there were 10 (47.62%, 10 of 21) cases (patients 1, 2, 3, 4, 8, 11, 12, 17, 19, and 20) of hemorrhage. After the treatment of transfusion or expansion of blood volume, patients with hemorrhage got good recovery, and none of these patients displayed circulatory or coagulation disorders (Table 2).

**Table 2:** Peri- and post-operative complications and managements

Complications	Cases (*n*/%)	Management measures	Outcomes
Hemorrhage	10/47.62%	Autologous or allogeneic transfusion, and expansion of blood volume	All got good recovery.
Cerebrospinal fluid leakage	5 (23.81%)	Two patients having a lesion on the lateral side of the dura were continuous sutured.	All wounds were healed.
		Another 3 patients having leakage in front of the dura were blocked lesions with fibrin glue or plugged with a gelatin sponge prior to the extubation of the drainage tubes 1 to 2 weeks later after the surgery.	
Intercostal nerve palsy	4 (19.05%)	Administration of dehydrating agents, hormones, and neurotrophic drugs	Got complete remission 3–6 months later.
Neurological deterioration	3 (14.29%)	Administration of neurotrophic drugs and hormones, and rehabilitation-oriented exercise	Two patients were improved but 1 not.
Superficial infection	1 (4.76%)	Dressing change and debridement	Wound healed smoothly.

Five (23.81%, 5 of 21) patients (patients 1, 8, 12, 15, and 17) developed cerebrospinal fluid leakage resulting from iatrogenic injury of the dural sac. Specifically, the condition was related to the strong adhesion between the dura and decompressed PLL, which caused injury upon their surgical separation. Among these patients, two had lesions on the lateral side of the dura, which was continuously sutured, and the other three had leakage in front of the dura, which was blocked with fibrin glue or plugged with a gelatin sponge before the extubation of the drainage tubes 1 to 2 weeks later after the surgery. All patients exhibited wound healing at an expected rate (Table 2).

Four (19.05%, 4 of 21) patients (patients 4, 8, 10, and 15) developed intercostal nerve palsy and all manifested numbness in the nerve root dominant range, which was possibly caused by surgical pulling or cutting. These patients were administered with dehydrating agents, hormones (dexamethasone), and neurotrophic drugs (mecobalamin) and exhibited complete remission 3–6 months later after the operation (Table 2).

Three (14.29%, 3 of 21) patients (patients 4, 8, and 11) exhibited postoperative neurological deterioration, two cases of which were considered to be caused by spinal cord ischemia-reperfusion injury and one by surgical manipulation. They received nerve nourishment, hormone therapy, and rehabilitation-oriented exercise. In the final follow-up visit, two patients (patients 4 and 8) showed an improvement in neurologic function, whereas one (patient 11) did not show improvement (Table 2).

Lastly, one (patient 3, 4.76%) of 21 patients was found incision-caused superficial infection. No deep infection was found. The patient was subject to dressing change and debridement and exhibited smooth wound healing (Table 2).

## Discussion

Posterior circumferential decompression and pedicle-screw internal fixation is considered to exhibit advantages in the treatment of TOPLL, such as direct decompression, avoid of damaging the intrathoracic tissues, and reconstruction of spinal stability. This surgical procedure has been shown to be an effective therapy and achieves satisfactory clinical outcomes for patients with properly selected signs, nevertheless, this approach is associated with a certain rate of complications [3, 4, 11, 12] In this study, 12 of the 21 patients were found with 23 complications (mainly short-term complications) including 10 cases of intraoperative hemorrhage, 5 of cerebrospinal fluid leakage, 4 of intercostal nerve palsy, 3 of neurological deterioration, and 1 of superficial infection. Of note, hemorrhage was defined as a kind of complication in this study while not in some other studies (e.g., References [4, 6]). If hemorrhage (in 10 cases) was excluded, there would be 13 complications in a cohort of 21 patients in our study. The complication rate in our study is actually much lower than in some other similar studies involving posterior circumferential depression for patients with TOPLL. For example, Takahata et al. reported 25 cases of postoperative cerebrospinal fluid leaks, postoperative neurologic deterioration, or deep infection in 30 patients with TOLL [6]. Li et al. showed 8 cases of postoperative cerebrospinal fluid leakage, postoperative paralysis, or infection in 7 patients [13]. Hu et al. indicated 19 cases of postoperative neurological deterioration or cerebrospinal fluid leakage in 26 patients [4]. Liu et al. demonstrated 32 cases of postoperative cerebrospinal fluid leakage, neurological deterioration or infection in 35 patients [3]. This might be related to the fact that these surgical procedures in our study were performed later (between February 2010 and December 2014) and thus the techniques might be further developed and surgeons mastered more experience compared with previous studies (e.g., References [4, 6, 13]). In addition, to decrease any possible unfavorable surgical outcomes, associated with decompression in multiple vertebral levels [6], circumferential decompression was controlled within 3 segments of the thoracic vertebral levels in this study. This might be another reason for the lower complication rate in this study in comparison with other previously reported.

### Intraoperative hemorrhage

Intraoperative hemorrhage (defined as blood loss above 1500 ml) is commonly complicated with posterior circumferential decompression for patients with TOPLL. In this study, 10 of 21 (47.62%) patients were found intraoperative hemorrhage. Intraoperative blood loss is related to surgical time and surgeons’ experience. According to our experience, during the early stages of surgeries, patients had more blood loss. However, with the accumulation of surgeons’ experiences and skills, blood loss tended to be reduced. The average operation time of 4.0 h (240 min) and mean blood loss of 1619 ml in our study were lower than in some other studies such as Takahata et al.’s and Hu et al.’s studies (average operation time 389 and 279 min, respectively; and mean blood loss 1883 and 2257 ml, respectively) [4, 6].

Blood loss appeared mainly due to two reasons. First, when the vertebral canal was opened, the stenotic spinal canal might be seen considerable bleeding because of the tortuous spinal venous plexus. Common prone position increases the abdominal pressure and thus decreases blood reflux. This might lead to vertebral venous congestion and in turn increases the intraoperative blood loss. As such, patients should be additionally hanging abdomen with a lower head and buttocks. In addition, posterior laminectomy should be performed to completely remove the lamina instead of opening it in a worm-eaten fashion, which is followed by temporary oppression with hemostatic gauze or a gelatin sponge for several minutes until bleeding is minimized so that the next step could be performed. Second, during the separation process of the PLL, damage to the intercostal arteries and veins would lead to considerable blood loss. Hence, flexible application of bipolar coagulation is recommended during the operation. In particular, bipolar coagulation around the pedicle can achieve significant hemostasis. In addition, intercostal artery can be ligated in advance, but in principle, such ligation should not be performed over 3 segments. For patients suffering hemorrhage, it is recommended estimating the potential volume of blood loss and thus preparing sufficient blood (allogenic blood). Another option is to use autologous transfusion to reduce massive allogenic blood transfusions. In addition, a controllable lowering of blood pressure may be adopted during the operation to limit bleeding, but the systolic pressure should not be below 100 mmHg. After the blood transfusion or expansion of blood volume, all patients in this study got good recovery.

### Cerebrospinal fluid leak

In this study, 5 of 21 (23.81%) patients had dural ruptures and cerebrospinal fluid leaks that were due to iatrogenic dural sac injury. This rate is lower than 38.5% (10 of 26 patients) in Hu et al.’s study [4], 40% (12 of 30 patients) in Takahata et al.’s study [6], 57.1% (4 of 7 patients) in Li et al.’s study [13], and 63% (22 of 35 patients) in Liu et al.’s study [3]. The main preventative measures are as follows: First, upon the separation of the PLL from the dura, a nerve dissector should be used to operate step by step from an edge with relatively apparent boundary and gradually to the area with severe lesions. Second, during the above separation process, it is necessary to avoid forcible pulling. Although usually beak-type ossification of the posterior longitudinal ligament (OPLL) can be directly separated and resected, for OPLL which was relatively large and adhered to the dural adhesions, it was not required to completely resect it so as to avoid the resulting dural taring and cerebrospinal fluid leak and other complications. Instead, we tried to make it suspending. In the inured spinal segments, we used a burr to remove 1/3 of the back of the vertebral body from the both sides of the posterior wall of the vertebral column, which was then passed through the two sides. Sometimes, 1–2 intercostal blood vessels or nerves were ligated, and the spinal cord and PLL were slightly pulled to one side using the ligation thread to increase the surgical focus. OPLL was gradually burred to become thinning and suspending, which assured complete decompression. Third, during the process of grinding the ossified PLL, a stable support is needed because the swing of the drill may also directly injure the dura. Fourth, if the dura is accidently torn, it should be subjected to edge locking and continuous suture on one side or blocking with fibrin glue or a gelatin sponge for a frontal tear. These above measures may decrease the occurrence of cerebral fluid leak. Fifth, 1–2 weeks later instead of 2 days later as usual after the operation, patients were extubated the drainage tubes, sutured the drainage port, and got pressure bandaging of the wound.

### Intercostal nerve paralysis

In Li et al.’s study, there was only 1 case of postoperative paralysis among 7 patients [13], and in some other studies [3, 6], no postoperative paralysis was found. In contrast, there was higher rate (19.05%, 4 of 21 patients) of intercostal nerve paralysis in this study. Among the patients with intercostal nerve paralysis, 2 resulted from nerve root pulling and 2 from surgical cutting, but all exhibited complete remission within one year. To prevent such conditions, manipulation should be gentle during the operation. Specifically, when drill is used on the facets, it should be operated along the orientation of the nerve root and its angle entering the nerve root should be minimized so as to decrease the possibility of nerve root injury. Indeed, there was one case of intercostal nerve paralysis occurred when nerve root was exposed and was drilled underneath, causing the nerve root winded along the drill and thus damaged. As such, it is recommended that drilling manipulation should be carried out on the shoulder of the nerve root following its orientation. In addition, if the nerve root generates too much pulling force on the spinal cord, it can be cut. Nonetheless, it is recommended that no more than three nerve roots should be cut. For this reason, the nerve roots were cut in 2 patients in this study. Since intercostal nerves are mainly sensory branches, the functions were rehabilitated 3 months later and within 1 year after the operation in this study.

### Neurologic deterioration

In some previous reports, for patients with TOPLL after the posterior circumferential decompression, the rate of neurologic deterioration was 29% (10 of 35 patients) [3], 33% (10 of 30 patients) [6], and 34.6% (9 of 26 patients) [4]. In this study, 3 of 21 patients (14.29%) developed postoperative neurological deterioration, including 2 suffered from spinal cord ischemia-reperfusion injury, whereas the other from manipulation-induced spinal cord trauma. The rate in this study is lower than in the above studies. To avoid the occurrence of such complications, the following suggestions are recommended. First, a large dose of methylprednisolone needs to be administered during the operation so as to minimize the intraoperative spinal cord edema and post-decompression spinal cord injury associated with ischemia-reperfusion. Second, intraoperative neurologic functions reflected by somatosensory evoked potentials need to be monitored. Third, the dynamic instant intraoperative blood loss should be limited to less than 400 ml, thereby both ensuring blood supply for the spinal cord and preventing excessive reduction of intraoperative blood pressure. Fourth, when the posterior lamina is opened, the intact bone plate, including the bilateral and medial sides of the facet and lamina, should be completely removed (i.e., laminectomy) to minimize the possibility of spinal cord injury. Fifth, while a drill or rongeur is used to clean the remaining part of the facet or pedicle, the apparatus should be kept away from the spinal cord. In addition, during the operation, the opposing side of the spine should be temporally fixed (prefixed) to avoid unnecessary shock to the spinal cord. Sixth, for the excision of the ossified PLL, it is recommended gradually removing it from the upper and lower ends of the ossified region, without resorting to violent tearing using a rongeur. Lastly, when a drill is used, both elbows of the surgeons should be rested on a solid support to avoid substantial swing.

### Superficial infection

In contrast to the involvement of deep infection or internal organ infection in some other studies [4, 6, 13], we found only 1 of 21 patients (4.76%) developed a superficial infection, which is possibly associated with the over-application of bipolar coagulation or a protracted operation. Our preventative measure is to administer antibiotics at 30 min prior to the operation, which is repeated if the surgical duration is above 4 h. During the operation, cutaneous or subcutaneous administration of bipolar coagulation should be performed in a punctate fashion to avoid ischemia resulted from flaky homeostasis. If superficial infection occurs, treatments mainly comprise dressing change and debridement, which generally leads to satisfactory healing.

Generally, although the complications from posterior circumferential decompression for TOPLL were referred in some previous reports, they were mainly described briefly and technically, and the surgery-associated complications had been rarely systematically summarized. We herein reported the outcomes, and complications and related managements of patient with TOPLL who received posterior circumferential decompression. Particular, we systematically summarized the posterior circumferential decompression-associated complications and the causes and corresponding preventive and treating measures, which have been rarely reported. This study will help prevent and treat the surgical complications during posterior circumferential decompression for TOPLL.

There are some limitations in this study. This is a retrospective study that might bring about bias of patient selection and incompleteness of some important medical information of patients. In addition, the follow-up period is not long. Next, more patients will be recruited to receive posterior circumferential decompression and subject to longer follow-up examination to validate these results.

## Conclusions

In summary, posterior circumferential decompression and pedicle-screw internal fixation is effective for the treatment of TOPLL but is also associated with some complications. Therefore, it is necessary to proactively prevent and treat the resulting complications. With proper management measures, most complications can be treated.

## Abbreviations

CT: Computed tomography
JOA: Japanese Orthopaedic Association
MRI: Magnetic resonance imaging
PLL: Posterior longitudinal ligament
TOPLL: Thoracic ossification of posterior longitudinal ligament
